# The formation of cariogenic plaque to contemporary adhesive restorative materials: an in vitro study

**DOI:** 10.1007/s10266-024-00913-5

**Published:** 2024-03-19

**Authors:** Anna Lehrkinder, Olivia Rydholm, Anna Wänström, Keisuke Nakamura, Ulf Örtengren

**Affiliations:** 1 Department of Cariology, Institute of Odontology, Sahlgrenska Academy, University of Gothenburg, Box 450, 405 30 Göteborg, Sweden; 2 Department of Advanced Free Radical Science, Tohoku University Graduate School of Dentistry, Sendai, Japan; 3 Department of Material Science and Technology, Faculty of Odontology, Malmö University, Malmö, Sweden

**Keywords:** Caries prevention, Biofilm, Dental materials, Fluoride, Ions

## Abstract

The research exploiting the ability of dental materials to induce or prevent secondary caries (SC) development still seems inconclusive. Controlling bacterial adhesion by releasing bacteriostatic ions and improving the surface structure has been suggested to reduce the occurrence of SC. This paper analyses the impact of five distinctively composed dental materials on cariogenic biofilm formation. Forty-five specimens of three composites (CeramX Spectra ST, Admira Fusion, Beautifil II) and two glass–ionomers (Fuji II LC, Caredyne Restore), respectively, were incubated in bacterial suspension composed of *Streptococcus mutans*, *Lactobacillus acidophilus*, *Streptococcus mitis, Streptococcus sanguinis, and Streptococcus salivarius* at pH 7.0 and 5.5. Coverslips were used as a control. Adhered bacteria were collected after 2, 4, 6, 12, 24, and 48 h and analyzed using quantitative polymerase chain reaction (qPCR). Fluoride leakage was measured at each collection. The specimens’ surface topography was assessed using interferometry. In the present study, surface roughness seemed to have a partial role in bacterial adhesion and biofilm formation, together with chemical composition of the materials tested. Despite differences in fluoride leakage, biofilm accumulation was similar across materials, but the number of adhered bacteria differed significantly. A release of other ions may also affect adhesion. These variations suggest that certain materials may be more prone to initiating secondary caries.

## Introduction

Even though the individual caries risk is regarded as the main factor for development of secondary caries, dental restorative materials such as composite resin-based material (hereafter referred to as composite) have also been suggested to be associated with the development of secondary caries [1]. Studies have reported shorter longevity and higher failure rates among composites compared to amalgam [2–7]. Secondary caries is reported to be one of the most common reasons for registered failure of composite restorations [2, 8–12].

Various factors have been suggested to why secondary caries seems to appear more common in conjunction with composite restorations. Microgaps causing microleakage have for a long time been associated with development of secondary caries [13–15]. Still, Jokstad [16] found no evidence for a correlation between microleakage and development of secondary caries clinically and at present no consensus on the subject is achieved [1].

Composite has been reported to be more receptive to plaque accumulation than enamel and other restorative materials [17–19]. In comparison, glass–ionomer materials have been reported to accumulate fewer mutans streptococci [20]. Surface roughness, as well as the surface-free energy, can influence bacterial adhesion [21–23]. The bacteria seem to have the ability to change the surface topography of the material, resulting in further facilitated adherence [24]. In addition, the chemical composition of the material may also influence adhesion and composition of the biofilm [17]. In that respect, the composition and the texture of the surface is important, in particular the type of filler used (e.g., silicon vs. carbon content), for formation of a pellicle favouring initial bacterial colonization [22, 25, 26].

Formation of biofilm on the surface of the tooth starts with formation of the pellicle within minutes after exposure to saliva [26]. The early bacterial colonizers include *Actinomyces* and some streptococci (i.e., *Streptococcus sanguinis, Streptococcus oralis, and Streptococcus mitis*) while *Streptococcus mutans* is not found in large amounts in the initial biofilm [27–29].

Since the development of secondary caries often occurs on approximal surfaces, which are found difficult to maintain good oral hygiene, the anti-caries features of the restorative materials can be decisive. Regarding composite material, substances have been reported to be eluted from the material as a result of an incomplete polymerization [30, 31]. Some of these compounds have been shown to have bacteria-stimulating effects [32]. Still, other studies have shown that added components such as boron and strontium in composites might have an antimicrobial effect [33–35]. It is likely to assume that more materials with these properties within the near future will be launched [17, 36].

Glass ionomer cements have also been reported to have a secondary caries-inhibiting effect due to fluoride release [37]. Fluoride is supposed to act as an anti-caries agent in different manners. The most evident effect is its ability to form deposits of CaF_2_ from topical fluoride solutions on the tooth surface which counteracts demineralization and facilitates remineralization by formation of fluorapatite [38, 39]. Fluoride can also interfere with the glycolytic metabolism and acid production of cariogenic bacteria by direct bond or in combination with metal ions, to enzymes [40]. Of importance is the fluoride’s ability to enter the bacterial cell as HF, acting as a weak acid when dissolved in water [41]. Inside the cell with its higher pH, the acid is dissolved as H^+^ and F^−^ followed by accumulation of H^+^ reducing the proton gradient and enzyme activity. An increased amount of F^−^ will inhibit glycolytic enzymes and proton extruding pumps, resulting in inhibition of carbohydrate metabolism and acid production [40, 42]. Despite the suggested inhibition of bacterial metabolism, however, there is still no consensus regarding the anti-cariogenic effect of F^−^ [42].

Other ions [e.g., strontium (Sr^2+^), boron (B^3+^), zinc (Zn^2+^), silver (Ag^+^)] with remineralising and antimicrobial effects have been incorporated in fillers of dental composites [35]. Sr has shown positive effects in vitro due to conversion of hydroxyapatite into strontium apatite, resulting in increased acid resistance [11–13, 43, 44]. B has been suggested as effective in caries prevention due to its antimicrobial properties [45, 46]. Silver and zinc oxide particles have been shown to inhibit mutans streptococci and lactobacilli [47]. Still, the inhibitor effects of these ions on cariogenic bacteria have been questioned [48]. Yoshihara et al. found a change in surface topography in ion-releasing materials when subjected to an acid environment resulting in ingrowth of bacteria in pits and crevices at the surface. They concluded that the change in topography and the low concentration of ion leakage did not inhibit cariogenic bacteria [48].

Even though research has been performed on the ability of dental materials to induce or prevent secondary caries, the results obtained yet seem inconclusive and more research is therefore needed to increase the knowledge.

### Aim

The present study aimed to increase the knowledge by;

(i) investigating differences in formation of the cariogenic biofilm onto surfaces of five contemporary dental materials, depending on their composition, and (ii) evaluating the interrelation between products’ surface characteristics and biofilm formation.

The null hypotheses stated for the present study were as follows:

1. there was no difference in the number of adhered bacteria among different dental materials,
2. the chemical composition as stated by the manufacturer would not influence the composition of the biofilm,
3. the surface roughness of the materials tested would not have impact on the biofilm formation, and
4. the biofilm would not have an impact of the surface of the materials tested.

## Materials and methods

### Materials

Five contemporary dental materials for direct use were investigated in vitro. Detailed information about tested materials is presented in Table 1. Three composite materials and two polyalkeonate cements (1 resin-modified glass ionomer and 1 chemically cured zinc–glass–ionomer) were compared. The three composites were chosen based on their composition. CeramX Spectra ST(CE) is an ordinary composite. In contrast, Beautifil II (BE) is claimed by the manufacturer to be a bioactive material with ion-releasing filler and Admira Fusion (AD) is considered as an ormocer (i.e., inorganic–organic hybrid polymer).

**Table 1:** Materials tested in the present study and their ingredients as given by the manufacturer and their SDS respectively

Material	Abbr	Manufacturer	Colour	Batch number	Ingredients
CeramX Spectra ST	CE	Dentsply Sirona; Konstanz, Germany	A3	1906000651	Ethoxylated Bisphenol A Dimethacrylate, Urethane modified Bis-GMA dimethacrylate resin, 2,2ʹ-ethylenedioxydiethyl dimetharcylate, ytterbium trifluoride, 2,6-di-tert-butyl-p-cresol, pre-polymerized SphereTEC® fillers, non-agglomerated barium glass, ytterbium fluoride
Beautifil II	BE	Shofu; Kyoto, Japan	A3	11930	Bis-GMA, Triethylenglycol Dimethacrylate, Aluminofluoro-Borosilicate Glass, Al_2_O_3_, DL-Camphorquinone, Surface pre-reacted glass–ionomer filler, others
Admira Fusion	AD	VOCO; Cuxhaven, Germany	A3	1921324	Organically modified silicic acid, fumes silica, 2,6-di-tert-butyl-p-cresol
Fuji II LC	FU	GC; Tokyo, Japan	A2	1907162	2-Hydroxyethyl methacrylate, polybasic carboxylic acid urethane dimethacrylate, dimethacrylate, Ca–Al–Si–O_2_–F–glass
Caredyne Restore	CA	GC; Tokyo, Japan	CV	1902261	Fluoro-almino-silicate glass, Fluoro-zinc-silicate glass, pigment, distilled water, polyacrylic acid, polybasic acid
Control	Ctr	Sarstedt; Newton, USA	–	90U1311	Plastic, non-pyrogenic (< 0.06 EU/ml), non-cytotoxic

### Manufacturing of the specimens; composites

Forty-five circular specimens of each material were manufactured using a polytetrafluoroethylene (PTFE) mould and in accordance with recent studies [49, 50]. The specimens were 10 ± 0.5 mm in diameter, and 1.5 ± 0.5 mm in thickness. The dimensions were controlled using a digital Vernier Caliper 0–150 μm (Cocraft, Clas Ohlson, Insjön, Sweden). A PTFE tape with a thickness of 75 µm (Clas Ohlson, Insjön, Sweden) was placed at the bottom of the mould to facilitate removal of the specimen. Special care was taken to avoid crimps and to ensure a completely flat tape. The latter was changed between each sample. The material was placed in the mould using a Cavifil injector Compule Gun (Ivoclar/Vivadent, Schaan, Liechtenstein) and condensed with an LM Carver 76–77 SI Nyström III (LM-Instruments, Pargas, Finland). Polyethylene strips (Kerr-Hawe/Kerr Dental, Kloten, Switzerland) were used to protect the upper surface from oxygen inhibition, and a glass plate was placed on top to ensure an even and equivalent surface of all specimens when being cured with an LED light unit (Bluephase G2, Ivoclar, Schaan, Liechtenstein) for 20 s. The irradiance (1280 ± 20 mW/cm^2^) was controlled regularly (Bluephase meter, Ivoclar, Schaan, Liechtenstein). The specimens were not polished after polymerization.

### Manufacturing of the specimens, Fuji II LC, Caredyne Restore

For the same purpose and with the same routine as described above, a tape was placed at the bottom of the mould. The material was mixed according to the manufacturer’s instruction using a 3 M ESPE CapMix™ capsule mixing device (3 M, St. Paul, Minnesota, US) and placed into the PTFE mould with an applicator (3 M, St. Paul, Minnesota, US) for glass–ionomer capsules. The specimens were then manufactured and light-cured in the same manner as for the composite samples.

Regarding Caredyne Restore, the material was mixed and cured according to the manufacturer’s instructions.

### Method

After production, specimens of FU and CA were stored in 20 ml distilled water 23 ± 1 °C for 7 days to ensure a more complete acid–base reaction.

Directly before the experiment, all specimens were sterilized by autoclaving in 100 °C for 10 min (Dx-150, Systec, Wettenberg, Germany). After sterilization, all specimens were placed in sterilized, distilled water for 8 days (37 ± 1 °C) to further facilitate complete reaction of the resin-modified glass ionomer cement (RMGIC), take the post polymerization of the composite into account, avoid dehydration of the chemically cured glass ionomer cement (GIC), and ensure likewise treatment of the specimens.

Stimulated saliva was collected from four authors, pooled, and centrifuged at 4 °C, 5000 rpm for 20 min (Eppendorf, Hamburg, Germany). Supernatant was sterilized by filtration with a 0.22 µm sterile filter (ThermoScientific, Rochester, USA). After consultation with the experts at the institute and approval from the head of institute, ethical approval was not considered necessary, since the authors own saliva was used. All specimens were dried and placed with the smooth side up in Petri dishes. They were all simultaneously submerged in sterilized saliva, to ensure even pellicle distribution, and incubated in 37 ± 1 °C for 1 h, to mimic the in vivo environment in the mouth as much as possible [51]. Meanwhile, 24-well, sterile plates (Sarstedt, Germany) were prepared by distributing 1 ml Brain Heart Infusion (BHI) broth (MERCK, Darmstadt, Germany) and 100 µl bacterial mix to each well. The bacterial suspension consisting of equal concentration of *S. mutans* IB*, S. mitis* ATCC 4956*, S. salivarius* CCUG 17825, *S. sanguinis* CCUG 10556, and *L. acidophilus* CCUG 5917 was prepared in BHI broth directly before the experiment. Separate bacteria strains were prepared in advance, incubated at 37 °C in anaerobic conditions. The optical density was measured and adjusted for each bacterial strain (550 nm; OD = 1.0), resulting in the final concentration of inoculum 1.3 × 10^5^ in the assay. The pH of the autoclaved broth was adjusted with 0.05 M HCl followed by filter sterilization to assure the sterile condition. The pH was adjusted to 5.5 in 50% of the wells to model the cariogenic situation. The pH in the remaining 50% of the wells was adjusted to 7.0, for a neutral pH.

**Fig. 1:**
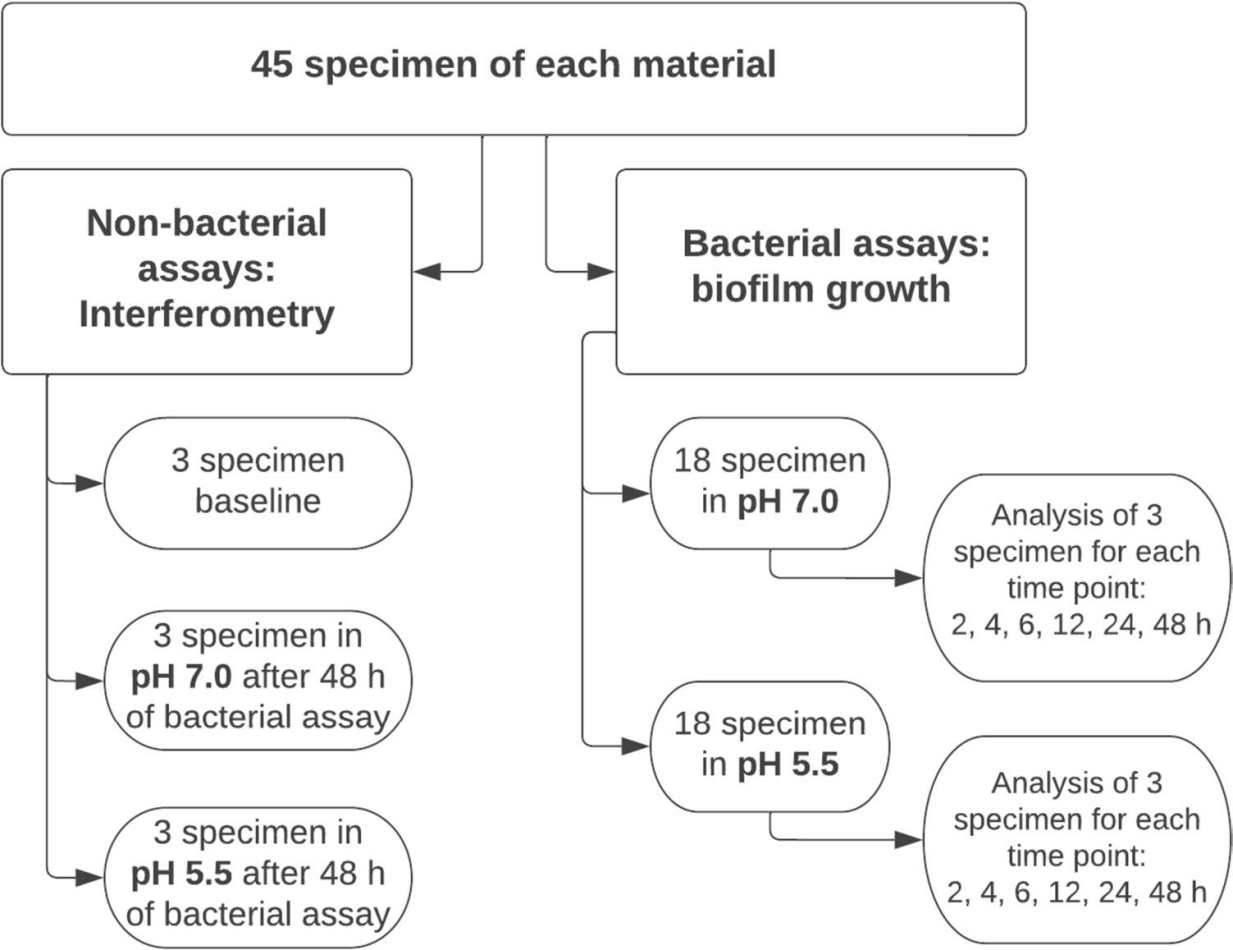
The flowchart illustrating material distribution and data collection in the study

The specimens were allocated as shown in Fig. 1. The specimens were rinsed from saliva with sterile, distilled water and placed in the wells with the smooth side up. Afterwards the plates were incubated at 37 ± 1 °C in anaerobic conditions for 48 h in total. Biofilm was collected from three specimens of each material at six different times; at 2, 4, 6, 12, 24, and 48 h. The broth pH was measured after the completion of the test to investigate any acid production by the bacteria. A pH electrode, Orion™ 8115BNUWP ROSS (ThermoFisherScientific™, Waltham, USA) was used for the measurement and was calibrated using standard buffer 4.0 and 7.0 (MERCK, Darmstadt, Germany) before use. Coverslips (Sarstedt, Newton, NC, USA) made of glycol-modified polyethylene terephthalate (PET-G) with a diameter of 13 mm, three for every collection time, were used as a control for adherence.

Sterile forceps were used to pick up the disc from the well. Each specimen was rinsed with 1 ml sterile water and tapped on sterile paper to remove the excess of planktonic bacteria. The biofilm was collected by wiping the surface using sterile collection paper-discs [52, 53] which directly afterwards were placed in Eppendorf tubes containing Tris–EDTA buffer pH 7.4 (TE buffer). TE buffer was used to solubilize and protect bacteria cells from degradation. Control samples were collected at each time by transferring 50 µl of the suspension into Eppendorf tubes, also containing TE buffer.

The collected biofilm was frozen (− 80 °C) until quantitative polymerase chain reaction (qPCR) analysis was performed. For analysis of surface topography changes, specimens incubated for 48 h were investigated. The specimens were cleaned from remaining bacteria or extracellular matrix using ultrasonic cleaning (Biosonic UC 125, Coltene/Whaledent GMbH, Langenau, Germany) in distilled water for 2 min, before analyzing the surface by interferometry. Three specimens kept in BHI broth without bacteria served as a negative control for topography changes and were analyzed at 48 h.

### Quantitative polymerase chain reaction (qPCR)

Prior to qPCR analysis, tubes with biofilm samples were placed in thermoshaker (TS-100C, Biosan, Latvia) for 10 min at 95 °C and 1000 rpm to release genomic DNA. The qPCR absolute quantification analysis was performed on magnetic induction cycle (MIC) analyser (Bio Molecular Systems, Upper Coomera, Australia). A conserved region (16S rRNA) in the ribosomal gene present in all bacteria was amplified using the universal primers to establish the count of all collected bacteria from the disc at each time point. Species-specific primers, previously described and validated [54–56], were used for amplification. Detailed information about primer sequence and assays is presented in Table 2. The reaction mixture of 20 µl in total contained: 1 × qPCRBIO SyGreen mix (PCR BioSystems, London, UK), 400 nM of each forward and reverse primers (Sigma-Aldrich, LLC), and 2.5 µl (< 1 µg genomic) DNA template. All amplifications were carried out as duplicates in MIC Tubes and Caps (BioMolecular Systems, Upper Coomera, Australia). All data were analyzed using MIC software (BioMolecular Systems, Upper Coomera, Australia). Standard curves for quantification of specific bacterial strains were constructed with known concentrations (tenfold dilution from 10^8^ to 10^1^ in ultrapure water) of genomic DNA extracted from reference strains.

**Table 2:** Primers’ sequence and qPCR conditions

	Primer’s sequence 5ʹ–3ʹ	Assay details
*Streptococcus mutans*	Forward: CTACACTTTCGGGTGGCTTG	95 ℃ 2 min,
[54]	Reverse: GAAGCTTTTCACCATTAGAAGCTG	40 × 95 ℃ 10 s, 61 ℃ 20 s, plate read
Total bacteria – universal	Forward: TGGAGCATGTGGTTTAATTCGA	94 ℃ 4 min,
[54]	Reverse: TGCGGGACTTAACCCAACA	40 × 94 ℃ 20 s, 62 ℃ 20 s, plate read
*Streptococcus salivarius*	Forward: GCAGCAGTAGCAGAGACGCT	95 ℃ 5 min,
[55]	Reverse: GTCATGACTTCTGCAGGCAC	40 × 95 ℃ 5 s, 60 ℃ 20 s, plate read
*Streptococcus mitis*	Forward: GTCGAAGGTGATGATATGAC	95℃ 3 min,
[55]	Reverse: CTGCATTCTGACGCATGACAG	40 × 95℃ 10 s, 55℃ 15 s, Plate Read
*Streptococcus sanguinis*	Forward: GTCGATGGCGAGGATCTAGAGC	95 ℃ 3 min,
[55]	Reverse: ACCTCAATCTCGCGAGCCGT	40 × 95 ℃ 10 s, 65 ℃ 30 s, plate read
*Lactobacillus acidophilus*	Forward: TGGAAACAGRTGCTAATACCG	98 ℃ 2 min,
[56]	Reverse: GTCCATTGTGGAAGATTCCC	40 × 98 ℃ 10 s, 62 ℃ 15 s, plate read

The number of detected bacteria (obtained from qPCR results) over time led to establishing Area Under the Curve (AUC) (GraphPad Software, San Diego, CA, USA) value for each material. To simplify the result interpretation, the AUC value for control was set to 1 (or 100%) and then the ratio:material AUC/control AUC was computed to indicate a growth above or below control level.

### Fluoride analysis

Fluoride concentration in broth was measured using the Orion 9609BNWP fluoride selective electrode (Thermo Fisher Scientific, Waltham, Mass., USA). To calibrate the electrode, three F standard solutions (0.1, 1.0, 10.0 ppm) (Thermo Fisher Scientific, Chelmsford, Mass., USA) were used. Total Ionic Strength Adjustment Buffer (TISAB III solution, ThermoScientific, Waltham, USA) was mixed with the sample in ratio 1:10 before measurement.

The first measurement was performed after the specimens were stored in water for 8 days. This was due to the different chemical reactions within the materials during the first period after manufacturing. Thereafter, the fluoride concentration was measured each time after the bacteria collection from the specimens.

### Interferometry

The surface topography was evaluated with a 3D optical profilometer using white light laser, gbs, smart WLI extended (Gesellschaft für Bild und Signal verarbeitung mbH, Immenau, Germany). A 50 × objective was used for all measurements. The data were evaluated with MountainsMap® Imaging Topography 7.4 (Digital Surf, Besancon, France) software. Surface roughness parameters were calculated after removing errors of form and waviness. A Gaussian filter with a size of 50 × 50 µm was used. The measuring area was 350 × 220 µm for all measurements. Three non-tested specimens of each material were measured on three areas, randomly distributed over the entire specimen, to gain a baseline of surface structure.

Three specimens from each material: without treatment, tested with and without bacteria after 48 h storage for both pH were measured on three areas, randomly distributed over the entire specimen.

The surface variation was described in height-, spatial-, and surface enlargement aspects in accordance with earlier studies [57]. Three parameters were selected; *S*_a_ that describes the average height distribution measured in µm, *S*_ds_ which is a measure of the density of summits over the measured area, measured in 1/µm^2^ and *S*_dr_ which describes the surface enlargement compared to a totally flat reference area, measured in %, a hybrid parameter that take account for variation in height and space.

### Statistics

The power analysis was done using G*Power3 software. Descriptive statistics (mean, standard deviation) were performed for all analysis (Microsoft Excel). For estimating differences in pH, number of bacteria between groups and surface parameters one-way ANOVA followed by Tukey test was used with a level of significance set to *p* < 0.05. R program (v3.6.3, R Core Team, Vienna, Austria) was used to create CLD (Compact Letter Display [58]. The GraphPad Prism Software (GraphPad Software, San Diego, CA, USA) was used to create a part of the artwork and analyses.

## Results

Table 3a and b displays the main findings of this study. The biofilm ratio is based on the control, which is set to 1.0. Denote letters describe significant difference (*p* < 0.05), and no significant difference is marked with the same letter.

**Table 3:** (a) Chemical and surface analysis comparison; (b) the influence on bacterial growth (values refer to growth (AUC_0–48 h_) ratio material/control)

(a)									
Grade	Fluoride ion release after 48 h (ppm) in pH 7.0	Fluoride ion release after 48 h (ppm) in pH 5.5	pH drop from 7.0	Surface analysis pH 7.0 with biofilm (mean)			Surface analysis pH 5.5 with biofilm (mean)		
				Sa (µm)	Sdr (%)	Sds (1/µm^2^)	Sa (µm)	Sdr (%)	Sds (1/µm^2^)
Highest	CA 12.4	CA 16.5	CE 1.54	CA^a^ 0.29	CA^a^ 50.76	Ctr^a^ 0.42	CA^a^ 0.26	CA^a^ 47.33	Ctr^a^ 0.38
	BE 10.4	BE 11.3	Ctr 1.50	CE^b^ 0.16	CE^b^ 6.52	CA^b^ 0.29	CE^b^ 0.13	CE^b^ 4.37	CA^b^ 0.26
	FU 9.1	FU 9.7	AD 1.40	FU^bc^ 0.11	FU^b^ 3.23	BE^c^ 0.22	FU^bc^ 0.09	FU^b^ 2.26	BE^bc^ 0.23
	CE 0.33	AD 0.33	BE 1.25	AD^cd^ 0.07	AD^b^ 2.09	AD^d^ 0.19	AD^c^ 0.06	BE^b^ 2.23	AD^cd^ 0.19
Lowest	AD 0.22	CE 0.29	FU 1.20	BE^cd^ 0.06	BE^b^ 2.09	CE^d^ 0.18	BE^bc^ 0.06	AD^b^ 1.75	CE^d^ 0.18
	Ctr (-)	Ctr (-)	CA 1.20	Ctr^d^ 0.05	Ctr^b^ 1.07	FU^d^ 0.17	Ctr^c^ 0.04	Ctr^b^ 0.62	FU^d^ 0.16

3b												
Grade	Biofilm pH 7.0 (AUC_0-48 h_ ratio/1)						Biofilm pH 5.5 (AUC_0-48 h_ ratio/1)					
	Total bacteria	*S.mutans*	*L.acidophilus*	*S.salivarius*	*S.mitis*	*S.sanguinis*	Total bacteria	*S.mutans*	*L.acidophilus*	*S.salivarius*	*S.mitis*	*S.sangius*
Lowest Highest	Ctr*^a^ 1	CA^a^ 2.08	Ctr*^a^ 1	Ctr*^a^ 1	Ctr^a^ 1	AD^a^ 1.02	BE*^a^ 1.01	FU*^a^ 4.31	Ctr*^a^ 1	Ctr*^a^ 1	Ctr^a^ 1	CE *^a^ 1.05
	AD*^b^ 0.40	AD^a^ 1.87	FU^b^ 0.62	AD *^b^ 0.45	AD^ab^ 0.85	Ctr^a^ 1	Ctr *^a^ 1	CA^b^ 2.99	FU^a^ 0.99	BE *^a^ 0.82	BE ^ab^ 0.54	Ctr^a^ 1
	BE*^b^ 0.40	FU *^a^ 1.83	CE^bc^ 0.40	BE*^b^ 0.36	FU^ab^ 0.57	CE*^a^ 0.82	FU *^ab^ 0.80	CE*^bc^ 2.31	CA^a^ 0.87	FU*^ab^ 0.61	CE^ab^ 0.54	BE*^a^ 0.90
	FU*^b^ 0.39	CE*^b^ 1.16	AD*^bc^ 0.37	FU*^b^ 0.34	CE^ab^ 0.52	BE*^a^ 0.61	CE*^bc^ 0.61	AD^cd^ 1.51	CE^ab^ 0.70	CE*^ab^ 0.48	AD^b^ 0.47	AD^a^ 0.86
	CE*^b^ 0.27	Ctr^b^ 1	BE^c^ 0.28	CE*^b^ 0.23	CA^ab^ 0.41	FU*^a^ 0.59	AD*^cd^ 0.51	BE^d^ 1.36	BE^bc^ 0.45	AD*^ab^ 0.44	FU^b^ 0.45	FU*^a^ 0.84
	CA*^b^ 0.25	BE^b^ 0.87	CA^c^ 0.21	CA*^b^ 0.22	BE^b^ 0.39	CA^a^ 0.47	CA*^d^ 0.29	Ctr^d^ 1	AD*^c^ 0.23	CA*^b^ 0.12	CA^b^ 0.43	CA^a^ 0.58

Denote letters describe significant difference (*p* < 0.05), no significant difference is marked with the same letter. Asterix marks significant difference in bacteria growth on individual material depending on tested pH

Total bacterial counts (Table 3b) in neutral pH on all tested materials were similar, with no significant differences among them, and it was significantly reduced in comparison to control. On the contrary, in the acidic pH, the differences in biofilm growth were significant among the materials, with the most considerable difference recorded between FU, BE *vs* CA and AD *vs* BE. The growth of *S. mutans* showed no significant difference or grew better on all materials when compared to control. While the growth of all other tested species was significantly reduced. Factors contributing to these differences were both pH and type of material. The growth of individual bacteria species was significantly influenced by pH (Table 3b).

**Fig. 2:**
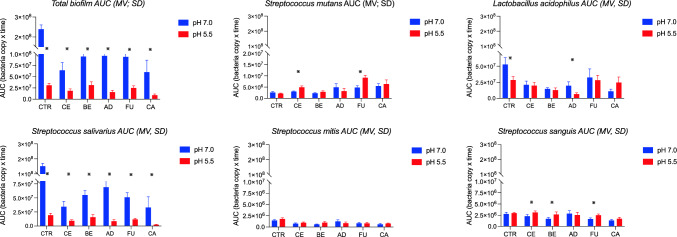
Comparison of bacteria growth (AUC_0–48 h_) in different pH conditions for all materials tested. Asterix marks difference in growth between different pH conditions (*p* < 0.05)

Difference was observed in growth of: total bacteria and *S. salivarius* on all tested materials, *S. mutans* on FU and CE; *L. acidophilus* on AD and control; *S. sanguinis* on FU and BE; *S. mitis* detection was similar on all materials regardless of pH (Fig. 2).

**Fig. 3:**
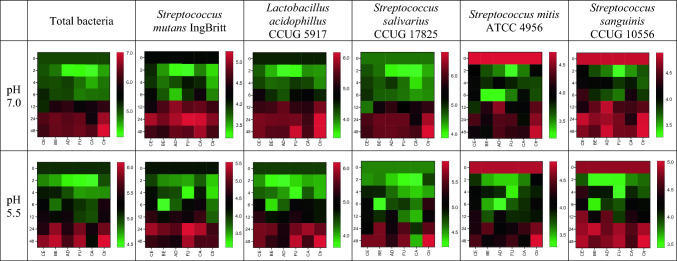
Bacterial concentration (log copy/10mm^2^, MV, *n* = 6), obtained at 0–48 h on Ceram.x (CE), Beautifil II (BE), Admira Fusion (AD), Fuji II LC (FU), Caredyne Restore (CA), and control (Ctr) in two pH conditions

Lactobacilli thrived best on FU (both tested pH) and least on both CA (pH 7.0) and AD (pH 5.5). The detection of all individual bacteria species in the biofilm formation on all tested materials, represented by corresponding AUC values and concentration, is presented in Figs. 2 and 3.

**Fig. 4:**
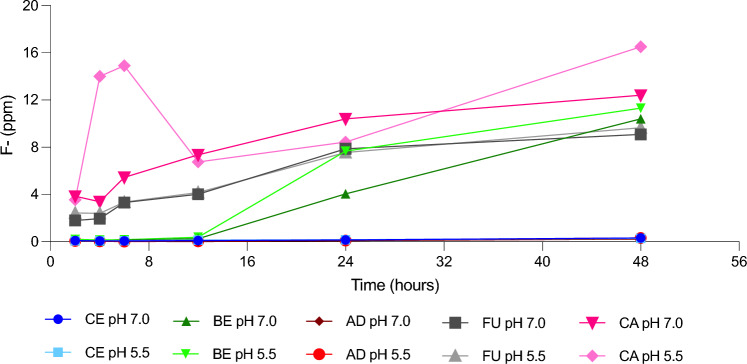
Fluoride leakage from discs during the microbiological experiment. Results presented in ppm (1 disc in 1 mL)

A high release of fluoride ions from the materials containing fluoride (CA, BE, FU; Fig. 4) did not influence the growth of cariogenic species (*S. mut*ans and *L. acidophilus*) (Table 3a and b). Other tested streptococci were more susceptible to all of the fluoride-releasing materials, regardless of pH, with most nominal growth observed on CA.

**Fig. 5:**
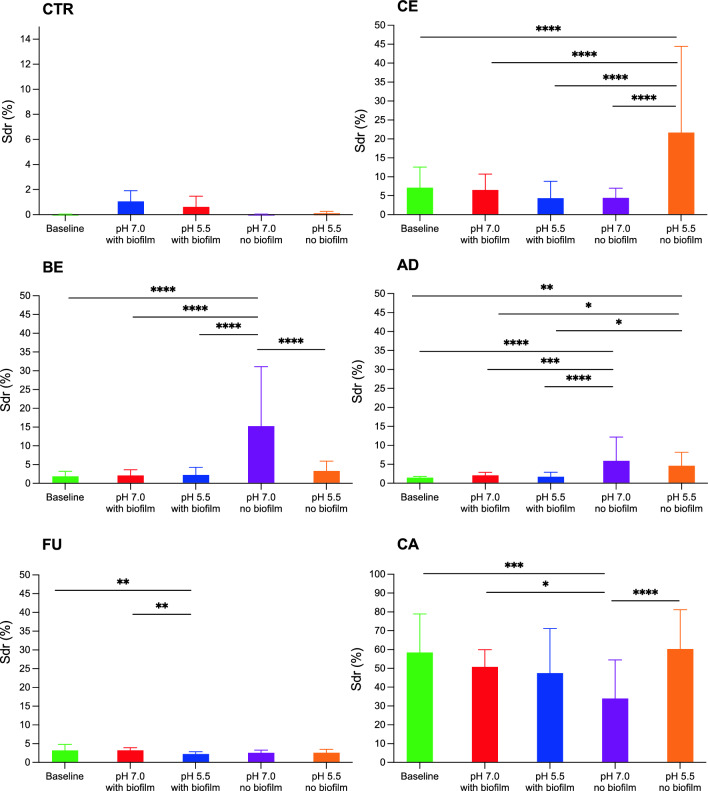
Significant differences in surface topography observed (Sdr) within the same material after 48 h in different environments. * = *p* < 0.05, ** = *p* < 0.01, *** = *p* < 0.001, **** = *p* < 0.0001

Significantly roughest surface (*S*_dr_) among tested materials, regardless of pH, was measured on CA; other values (*S*_a_ and *S*_ds_) differed significantly but to a limited extent (Fig. 5). The roughness of CA surface was greatly favoured by *S. mutans* in both pH environments. Smoother, with a comparatively low value of *S*_dr,_ BE attracted the least amount of *S. mutans* and *L. acidophilus* regardless of pH environment, while other species preferred it.

### Fluoride leakage

The analysis of fluoride leakage from discs kept in distilled water for 8 days prior to experiment revealed highest F^−^ release from FU (0.14 mg/g) followed by CA (0.03 mg/g) and BE (0.01 mg/g). However, during bacteriological exposure (48 h), CA and BE released more fluoride in both evaluated pH conditions (Fig. 4). The highest total fluoride leakage after 48 h was obtained from CA > BE > FU regardless pH.

### Surface analysis

Environment had a significant impact only on *S*_dr_ value (surface enlargement compared to totally flat reference area, %). Results of surface topology analysis are presented in Fig. 5.

## Discussion

The results of our study have shown that the number of adhered bacteria (both in total and the cariogenic strains) was affected with significant differences while grown on different tested materials, and therefore, the first null hypothesis was rejected.

Fluoride has in some studies affected bacterial growth [45, 59, 60]. That was not directly confirmed in the present study, since the bacterial growth pattern followed a similar trend on all materials despite the differences in fluoride leakage. The polyalkenoate cements reduced the total number of bacteria, even though the amount of *S. mutans* alone remained high. In this study, the ion-releasing BE decreased the cariogenic bacteria (*S. mutans, L. acidophilus*) growth despite the pH used. BE has also shown release of metal ions such as strontium and boron [61, 62], that together with a low *S*_dr_ value could explain the results. Therefore, the second null hypothesis concerning material composition was rejected.

Based on the results of this study, it is difficult to confirm or reject the third null hypothesis which states that surface roughness does not impact biofilm formation. We found that there were significant differences in roughness (Sdr) between materials but no significant difference in total amount of adhered bacteria in pH 7.0. In pH 5.5, FU and BE display a high biofilm adherence, despite having relatively smooth surfaces. CA displays low adherence of total amount of bacteria in pH 5.5, despite having a rough surface. In addition, the surface roughness measured in *S*_dr_ seem only partly to affect bacterial adhesion.

The fourth null hypothesis was partly accepted, as there was no significant difference in surface roughness within the tested restorative material, except from FU, when exposed to biofilm, compared to baseline.

The reliability of the applied methods was ensured with several measures.

The number of samples in each group was based on previous investigations with the assumption that all specimens of each material originated from a homogeneous population [50]. Additionally, sample size calculation was performed, based on a significance level of 5 and 80% power, to detect the difference in number of adhered bacteria over time (AUC) to the materials. The power calculation revealed that 2 samples are needed in each group; the study was performed on 3 samples, analyzed in duplicate to ensure high probability of detecting a significant difference.

Autoclaving instead of UV radiation was chosen for disc sterilization to minimize the risk of polymer degradation [63]. It can be speculated that the autoclaving with 100 °C for 10 min could increase the degree of conversion (DC). However, since all samples were accurately polymerized and the post-curing process (7 days in 37 °C water) was taken into account, autoclaving (100 °C, 10 min) would not increase the DC to a degree that should influence the result. The reason is that the time for autoclaving was too short to increase the DC in an already highly crossed network (ref. Prof Ulf Gedde, Department of Polymer Science, Royal Technical Institute, Stockholm, Sweden).

There is always a possibility that “eluded products”, e.g., monomers, can affect the bacterial presence. To decrease the risk of bias, the specimens were kept in distilled water for 7 days at 37 °C. It has been shown that significant leakage of the monomer occurs during the first 24 h to 7 days after polymerization [64, 65].

The specimens were coated in sterilized saliva to allow the pellicle formation to mimic the in vivo conditions in the oral cavity. To ensure an equal protein composition, saliva from four individuals was pooled before use and all specimens were coated simultaneously. In the production of the specimens, the LED light unit used for polymerization was calibrated by a Bluephase meter between every specimen, to ensure equal curing. The tip of the LED light unit had the same diameter as the specimens, which ensured an equal distribution of light. An equal curing may affect bacterial growth due to the risk of increased leaking of residual molecules [46]. Triplicates of specimens were used for each collection time and different pH. Additionally, a technical duplicate of each sample was run in the qPCR analyses in attempt to improve the reliability of the analyses with reduction of the influence of eventual biases [66]. Coverslips were used as a control by each time and specimens were kept in broth without bacteria for 48 h to serve as control when analyzing the surface. Three specimens were also used for each material, time, and pH in the interferometry. The measurements of surface roughness (*S*_dr_) were performed on three randomly distributed areas.

All the presented results showed that the number of bacteria was higher at baseline than after 2 h (Fig. 3). The reason for this is that at baseline, the bacteria were collected in the planktonic phase. At 2 h, they were collected as biofilm, as they just had started to adhere to the discs.

As stated, the present study focused on the total amount of bacteria, *S. mutans*, and *L. acidophilus.* These two aciduric and acidogenic bacterial species were chosen, because they are associated with the development of the caries disease. However, the microbiology of dental caries is not solely linked to these two species, but to the relationship between several different species, (Ecological Plaque Hypothesis) [67], hence why the study also focuses on the total amount of bacteria.

Hao et al. [17] and Zhang et al. [19] reported that dental composite seemed more receptive to plaque accumulation compared to glass–ionomers. Comparisons between data of different studies must be interpreted with caution, due to different test materials, methods, and analysis settings. The results from the present study both agree and disagree with these findings [17, 19]. In pH 7.0, there was no significant difference in accumulated biofilm between the tested materials, which disagrees with earlier findings [17, 19]. In pH 5.5 BE (a composite), and FU (a light curing glass ionomer) accumulated significantly more biofilm and CA (a chemically cured glass ionomer like material) less biofilm. The difference in biofilm formation is in agreement with earlier results [17, 19].

Regarding formation of cariogenic bacteria on glass ionomers, the results of the present in vitro study do not correlate with earlier findings, as significantly more *S. mutans* from glass ionomers was collected in pH 7.0 (CA) and pH 5.5 (FU) than from composites. Svanberg et al. [20] reported that glass–ionomer materials accumulate fewer *S. mutans* when compared to composite. However, the latter was conducted as an in vivo study and the type of glass ionomer tested was 3 M ESPE Ketac Silver (3 M, St. Paul, Minnesota, US), which is a silver-reinforced**,** chemically curing glass ionomer. Silver has an antibacterial effect that may explain the results of the recent study [10]. Glass–ionomer cement has been reported to have a secondary caries-inhibiting effect due to the releasing of fluoride [37]. The findings of the present study cannot counteract this, as the anti-caries effect of fluoride leakage in terms of counteracting demineralization and facilitating remineralization, was not investigated in this study. The growth pattern of cariogenic bacteria over time could be applied to the materials with high fluoride leakage rates. Release of fluoride ions has been claimed to have a bacteriostatic effect [45, 59]. Still, in the present study, the release of fluoride seemed not to influence the growth of *S. mutans* and *L. acidophilus* in particular. The finding agrees with the results of Poggio et al. [68]. They reported no reduction in the adhesion of *S. mutans* on glass–ionomers. Furthermore, the same study reported that glass–ionomers and flowable composites statistically showed greater adhesion values of *S. mutans* than did composites [68]. Still, data seem limited regarding adhesion properties of cariogenic bacteria on fluoride-releasing materials over an extended period and further studies seems needed.

Saku et al. [61] have shown composite containing pre-reacted glass ionomer filler particles, BE, to accumulate less biofilm in vivo and less *S. mutans *in vitro compared to two commercial types of composites. The latter result corresponds well to the findings of the present study with less cariogenic bacteria adhered to the surface of BE. The study of Saku et al. also concluded that there was no significant difference in antibacterial effect among the three composite resins tested [61]. However, only three subjects participated in the in vivo case, and the number of specimens used in the in vitro trial is not specified, so statistics and thereby results, can be questioned.

Some studies concluded that bacterial adhesion of *S. mutans* was not influenced by the surface roughness of the materials tested [68, 69]. However, no saliva was used in the study by Poggio et al. [68]. Other studies report that rougher surfaces bound more salivary proteins and, consequently, more bacteria [70, 71]. Even though the material CA showed the highest value of S_dr_, the significant differences in bacterial adhesion at different pH could not be displayed to any large extent.

Poggio et al. [68] states that “the hypothesis that the difference in bacterial adhesion [to different dental restorative materials] can be determined by the particular surface chemistry of the material itself as well as by different electrostatic forces between bacteria and restorative surfaces must be given serious consideration”. Further, Inoescu et al. [22] reported that after polishing, significantly less *S. mutans* biofilm formation was observed as a result of the modification of physical and chemical surface parameters of the resin‐based composites and stated that “these results indicate that the proportions of resin matrix and filler particles on the surface of resin‐based composites strongly influence *S. mutans* biofilm formation in vitro, suggesting that minimization of resin matrix exposure might be useful to reduce biofilm formation on the surface of resin‐based composites”. Interestingly, the same study also reported no difference in *S. mutans* biofilm formation on the surface of different resin‐based composites. However, considerations should be taken in interpretation, as a single‐species biofilm formation was investigated, without prior salivary pellicle formation [22]. The studies mentioned above could partly explain why the composite resin-based materials tested showed less adherence of cariogenic bacteria in pH 5.5.

It has been shown that bacteria can interfere with the surface of dental materials and change the topography, causing the surface to be rougher [24, 72]. In the present study, FU was the only material tested that showed a significant difference in surface roughness measured in S_dr_ after being kept in bacterial suspension for 48 h. However, it would have been interesting to investigate whether the outcome would have been different if measurements were performed after the specimens had been exposed to cariogenic biofilm during a longer time period.

Nedeljkovic et al. [1] concluded that secondary caries to composites could be associated with the properties of the material. However, the fact that the development of secondary caries also depended on the type and the location of the restoration indicates that other factors, such as surface properties, release of components, and lack of antibacterial properties, also had an influence. The results from the present study showed differences in adhesion of cariogenic bacteria between the tested materials. The material BE seemed to show less adherence compared to the others in both pH tested. It can be hypothesised that the reason is dependent on both the smooth surface, particular surface chemistry (not evaluated in this study), relatively high fluoride release together with release of other ions with known effect on cariogenic bacteria [61, 62]].

## Conclusions

Multiple factors seem to influence bacterial adhesion to dental restorative materials. The results of the present study have displayed differences between the materials tested, but it is difficult to precise the main factor for induction or prevention concerning bacterial adhesion, and thereby development of secondary caries. However, the present study showed that leakage of fluoride from the restorative material does not seem to have an inhibitory effect on bacterial adhesion to the materials tested. Still, it seems that the surface texture as well as the composition and leakage of other ions can affect adhesion of cariogenic bacteria.

## Data Availability

The paper contains all the data supporting this study's findings. The authors also provide detailed qPCR results upon request.
